# Supplementary material for: A Critical View of the Application of the APEX Software (Aqueous Photochemistry of Environmentally-Occurring Xenobiotics) to Predict Photoreaction Kinetics in Surface Freshwaters
Source: Molecules. 2019 Dec 18;25(1):9. doi: 10.3390/molecules25010009 (PMC7017383; doi:10.3390/molecules25010009)
Supplement: Supplementary file 1 [file molecules-25-00009-s001.zip › 3.2.4_gcc-4.4.0.zip!/3.2.4_gcc-4.4.0/3.2.4_gcc-4.4.0/doc/octave/html/liboctave/Matrix-and-Vector-Operations.html]

Matrix and Vector Operations - Octave C++ Classes


Next: Matrix Factorizations,
Previous: Arrays,
Up: Top


---

## 4 Matrix and Vector Operations

— : **Matrix** (void)  
— : **Matrix** (int r, int c)  
— : **Matrix** (int r, int c, double val)  
— : **Matrix** (const Array2<double> &a)  
— : **Matrix** (const Matrix &a)  
— : **Matrix** (const DiagArray<double> &a)  
— : **Matrix** (const DiagMatrix &a)

— : Matrix& **operator =** (const Matrix &a)

— : int **operator ==** (const Matrix &a) const  
— : int **operator !=** (const Matrix &a) const

— : Matrix& **insert** (const Matrix &a, int r, int c)  
— : Matrix& **insert** (const RowVector &a, int r, int c)  
— : Matrix& **insert** (const ColumnVector &a, int r, int c)  
— : Matrix& **insert** (const DiagMatrix &a, int r, int c)

— : Matrix& **fill** (double val)  
— : Matrix& **fill** (double val, int r1, int c1, int r2, int c2)

— : Matrix **append** (const Matrix &a) const  
— : Matrix **append** (const RowVector &a) const  
— : Matrix **append** (const ColumnVector &a) const  
— : Matrix **append** (const DiagMatrix &a) const

— : Matrix **stack** (const Matrix &a) const  
— : Matrix **stack** (const RowVector &a) const  
— : Matrix **stack** (const ColumnVector &a) const  
— : Matrix **stack** (const DiagMatrix &a) const

— : Matrix **transpose** (void) const

— : Matrix **extract** (int r1, int c1, int r2, int c2) const

— : RowVector **row** (int i) const  
— : RowVector **row** (char \*s) const

— : ColumnVector **column** (int i) const  
— : ColumnVector **column** (char \*s) const

— : Matrix **inverse** (void) const  
— : Matrix **inverse** (int &info) const  
— : Matrix **inverse** (int &info, double &rcond) const

— : ComplexMatrix **fourier** (void) const  
— : ComplexMatrix **ifourier** (void) const

— : DET **determinant** (void) const  
— : DET **determinant** (int &info) const  
— : DET **determinant** (int &info, double &rcond) const

— : Matrix **solve** (const Matrix &b) const  
— : Matrix **solve** (const Matrix &b, int &info) const  
— : Matrix **solve** (const Matrix &b, int &info, double &rcond) const

— : ComplexMatrix **solve** (const ComplexMatrix &b) const  
— : ComplexMatrix **solve** (const ComplexMatrix &b, int &info) const  
— : ComplexMatrix **solve** (const ComplexMatrix &b, int &info, double &rcond) const

— : ColumnVector **solve** (const ColumnVector &b) const  
— : ColumnVector **solve** (const ColumnVector &b, int &info) const  
— : ColumnVector **solve** (const ColumnVector &b, int &info, double &rcond) const

— : ComplexColumnVector **solve** (const ComplexColumnVector &b) const  
— : ComplexColumnVector **solve** (const ComplexColumnVector &b, int &info) const  
— : ComplexColumnVector **solve** (const ComplexColumnVector &b, int &info, double &rcond) const

— : Matrix **lssolve** (const Matrix &b) const  
— : Matrix **lssolve** (const Matrix &b, int &info) const  
— : Matrix **lssolve** (const Matrix &b, int &info, int &rank) const

— : ComplexMatrix **lssolve** (const ComplexMatrix &b) const  
— : ComplexMatrix **lssolve** (const ComplexMatrix &b, int &info) const  
— : ComplexMatrix **lssolve** (const ComplexMatrix &b, int &info, int &rank) const

— : ColumnVector **lssolve** (const ColumnVector &b) const  
— : ColumnVector **lssolve** (const ColumnVector &b, int &info) const  
— : ColumnVector **lssolve** (const ColumnVector &b, int &info, int &rank) const

— : ComplexColumnVector **lssolve** (const ComplexColumnVector &b) const  
— : ComplexColumnVector **lssolve** (const ComplexColumnVector &b, int &info) const  
— : ComplexColumnVector **lssolve** (const ComplexColumnVector &b, int &info, int &rank) const

— : Matrix& **operator +=** (const Matrix &a)  
— : Matrix& **operator -=** (const Matrix &a)

— : Matrix& **operator +=** (const DiagMatrix &a)  
— : Matrix& **operator -=** (const DiagMatrix &a)

— : Matrix **operator !** (void) const

— : ComplexMatrix **operator +** (const Matrix &a, const Complex &s)  
— : ComplexMatrix **operator -** (const Matrix &a, const Complex &s)  
— : ComplexMatrix **operator \*** (const Matrix &a, const Complex &s)  
— : ComplexMatrix **operator /** (const Matrix &a, const Complex &s)

— : ComplexMatrix **operator +** (const Complex &s, const Matrix &a)  
— : ComplexMatrix **operator -** (const Complex &s, const Matrix &a)  
— : ComplexMatrix **operator \*** (const Complex &s, const Matrix &a)  
— : ComplexMatrix **operator /** (const Complex &s, const Matrix &a)

— : ColumnVector **operator \*** (const Matrix &a, const ColumnVector &b)  
— : ComplexColumnVector **operator \*** (const Matrix &a, const ComplexColumnVector &b)

— : Matrix **operator +** (const Matrix &a, const DiagMatrix &b)  
— : Matrix **operator -** (const Matrix &a, const DiagMatrix &b)  
— : Matrix **operator \*** (const Matrix &a, const DiagMatrix &b)

— : ComplexMatrix **operator +** (const Matrix &a, const ComplexDiagMatrix &b)  
— : ComplexMatrix **operator -** (const Matrix &a, const ComplexDiagMatrix &b)  
— : ComplexMatrix **operator \*** (const Matrix &a, const ComplexDiagMatrix &b)

— : Matrix **operator \*** (const Matrix &a, const Matrix &b)  
— : ComplexMatrix **operator \*** (const Matrix &a, const ComplexMatrix &b)

— : ComplexMatrix **operator +** (const Matrix &a, const ComplexMatrix &b)  
— : ComplexMatrix **operator -** (const Matrix &a, const ComplexMatrix &b)

— : ComplexMatrix **product** (const Matrix &a, const ComplexMatrix &b)  
— : ComplexMatrix **quotient** (const Matrix &a, const ComplexMatrix &b)

— : Matrix **map** (d\_d\_Mapper f, const Matrix &a)  
— : void **map** (d\_d\_Mapper f)

— : Matrix **all** (void) const  
— : Matrix **any** (void) const

— : Matrix **cumprod** (void) const  
— : Matrix **cumsum** (void) const  
— : Matrix **prod** (void) const  
— : Matrix **sum** (void) const  
— : Matrix **sumsq** (void) const

— : ColumnVector **diag** (void) const  
— : ColumnVector **diag** (int k) const

— : ColumnVector **row\_min** (void) const  
— : ColumnVector **row\_min\_loc** (void) const

— : ColumnVector **row\_max** (void) const  
— : ColumnVector **row\_max\_loc** (void) const

— : RowVector **column\_min** (void) const  
— : RowVector **column\_min\_loc** (void) const

— : RowVector **column\_max** (void) const  
— : RowVector **column\_max\_loc** (void) const

— : ostream& **operator <<** (ostream &os, const Matrix &a)  
— : istream& **operator >>** (istream &is, Matrix &a)

— : **ColumnVector** (void)  
— : **ColumnVector** (int n)  
— : **ColumnVector** (int n, double val)  
— : **ColumnVector** (const Array<double> &a)  
— : **ColumnVector** (const ColumnVector &a)

— : ColumnVector& **operator =** (const ColumnVector &a)

— : int **operator ==** (const ColumnVector &a) const  
— : int **operator !=** (const ColumnVector &a) const

— : ColumnVector& **insert** (const ColumnVector &a, int r)

— : ColumnVector& **fill** (double val)  
— : ColumnVector& **fill** (double val, int r1, int r2)

— : ColumnVector **stack** (const ColumnVector &a) const

— : RowVector **transpose** (void) const

— : ColumnVector **extract** (int r1, int r2) const

— : ColumnVector& **operator +=** (const ColumnVector &a)  
— : ColumnVector& **operator -=** (const ColumnVector &a)

— : ComplexColumnVector **operator +** (const ColumnVector &a, const Complex &s)  
— : ComplexColumnVector **operator -** (const ColumnVector &a, const Complex &s)  
— : ComplexColumnVector **operator \*** (const ColumnVector &a, const Complex &s)  
— : ComplexColumnVector **operator /** (const ColumnVector &a, const Complex &s)

— : ComplexColumnVector **operator +** (const Complex &s, const ColumnVector &a)  
— : ComplexColumnVector **operator -** (const Complex &s, const ColumnVector &a)  
— : ComplexColumnVector **operator \*** (const Complex &s, const ColumnVector &a)  
— : ComplexColumnVector **operator /** (const Complex &s, const ColumnVector &a)

— : Matrix **operator \*** (const ColumnVector &a, const RowVector &a)

— : ComplexMatrix **operator \*** (const ColumnVector &a, const ComplexRowVector &b)

— : ComplexColumnVector **operator +** (const ComplexColumnVector &a, const ComplexColumnVector &b)

— : ComplexColumnVector **operator -** (const ComplexColumnVector &a, const ComplexColumnVector &b)

— : ComplexColumnVector **product** (const ComplexColumnVector &a, const ComplexColumnVector &b)

— : ComplexColumnVector **quotient** (const ComplexColumnVector &a, const ComplexColumnVector &b)

— : ColumnVector **map** (d\_d\_Mapper f, const ColumnVector &a)  
— : void **map** (d\_d\_Mapper f)

— : double **min** (void) const  
— : double **max** (void) const

— : ostream& **operator <<** (ostream &os, const ColumnVector &a)

— : **RowVector** (void)  
— : **RowVector** (int n)  
— : **RowVector** (int n, double val)  
— : **RowVector** (const Array<double> &a)  
— : **RowVector** (const RowVector &a)

— : RowVector& **operator =** (const RowVector &a)

— : int **operator ==** (const RowVector &a) const  
— : int **operator !=** (const RowVector &a) const

— : RowVector& **insert** (const RowVector &a, int c)

— : RowVector& **fill** (double val)  
— : RowVector& **fill** (double val, int c1, int c2)

— : RowVector **append** (const RowVector &a) const

— : ColumnVector **transpose** (void) const

— : RowVector **extract** (int c1, int c2) const

— : RowVector& **operator +=** (const RowVector &a)  
— : RowVector& **operator -=** (const RowVector &a)

— : ComplexRowVector **operator +** (const RowVector &a, const Complex &s)  
— : ComplexRowVector **operator -** (const RowVector &a, const Complex &s)  
— : ComplexRowVector **operator \*** (const RowVector &a, const Complex &s)  
— : ComplexRowVector **operator /** (const RowVector &a, const Complex &s)

— : ComplexRowVector **operator +** (const Complex &s, const RowVector &a)  
— : ComplexRowVector **operator -** (const Complex &s, const RowVector &a)  
— : ComplexRowVector **operator \*** (const Complex &s, const RowVector &a)  
— : ComplexRowVector **operator /** (const Complex &s, const RowVector &a)

— : double **operator \*** (const RowVector &a, ColumnVector &b)

— : Complex **operator \*** (const RowVector &a, const ComplexColumnVector &b)

— : RowVector **operator \*** (const RowVector &a, const Matrix &b)

— : ComplexRowVector **operator \*** (const RowVector &a, const ComplexMatrix &b)

— : ComplexRowVector **operator +** (const RowVector &a, const ComplexRowVector &b)  
— : ComplexRowVector **operator -** (const RowVector &a, const ComplexRowVector &b)

— : ComplexRowVector **product** (const RowVector &a, const ComplexRowVector &b)  
— : ComplexRowVector **quotient** (const RowVector &a, const ComplexRowVector &b)

— : RowVector **map** (d\_d\_Mapper f, const RowVector &a)  
— : void **map** (d\_d\_Mapper f)

— : double **min** (void) const  
— : double **max** (void) const

— : ostream& **operator <<** (ostream &os, const RowVector &a)

— : **DiagMatrix** (void)  
— : **DiagMatrix** (int n)  
— : **DiagMatrix** (int n, double val)  
— : **DiagMatrix** (int r, int c)  
— : **DiagMatrix** (int r, int c, double val)  
— : **DiagMatrix** (const RowVector &a)  
— : **DiagMatrix** (const ColumnVector &a)  
— : **DiagMatrix** (const DiagArray<double> &a)  
— : **DiagMatrix** (const DiagMatrix &a)

— : DiagMatrix& **operator =** (const DiagMatrix &a)

— : int **operator ==** (const DiagMatrix &a) const  
— : int **operator !=** (const DiagMatrix &a) const

— : DiagMatrix& **fill** (double val)  
— : DiagMatrix& **fill** (double val, int beg, int end)  
— : DiagMatrix& **fill** (const ColumnVector &a)  
— : DiagMatrix& **fill** (const RowVector &a)  
— : DiagMatrix& **fill** (const ColumnVector &a, int beg)  
— : DiagMatrix& **fill** (const RowVector &a, int beg)

— : DiagMatrix **transpose** (void) const

— : Matrix **extract** (int r1, int c1, int r2, int c2) const

— : RowVector **row** (int i) const  
— : RowVector **row** (char \*s) const

— : ColumnVector **column** (int i) const  
— : ColumnVector **column** (char \*s) const

— : DiagMatrix **inverse** (void) const  
— : DiagMatrix **inverse** (int &info) const

— : DiagMatrix& **operator +=** (const DiagMatrix &a)  
— : DiagMatrix& **operator -=** (const DiagMatrix &a)

— : Matrix **operator +** (const DiagMatrix &a, double s)  
— : Matrix **operator -** (const DiagMatrix &a, double s)

— : ComplexMatrix **operator +** (const DiagMatrix &a, const Complex &s)  
— : ComplexMatrix **operator -** (const DiagMatrix &a, const Complex &s)

— : ComplexDiagMatrix **operator \*** (const DiagMatrix &a, const Complex &s)  
— : ComplexDiagMatrix **operator /** (const DiagMatrix &a, const Complex &s)

— : Matrix **operator +** (double s, const DiagMatrix &a)  
— : Matrix **operator -** (double s, const DiagMatrix &a)

— : ComplexMatrix **operator +** (const Complex &s, const DiagMatrix &a)  
— : ComplexMatrix **operator -** (const Complex &s, const DiagMatrix &a)

— : ComplexDiagMatrix **operator \*** (const Complex &s, const DiagMatrix &a)

— : ColumnVector **operator \*** (const DiagMatrix &a, const ColumnVector &b)

— : ComplexColumnVector **operator \*** (const DiagMatrix &a, const ComplexColumnVector &b)

— : ComplexDiagMatrix **operator +** (const DiagMatrix &a, const ComplexDiagMatrix &b)  
— : ComplexDiagMatrix **operator -** (const DiagMatrix &a, const ComplexDiagMatrix &b)

— : ComplexDiagMatrix **product** (const DiagMatrix &a, const ComplexDiagMatrix &b)

— : Matrix **operator +** (const DiagMatrix &a, const Matrix &b)  
— : Matrix **operator -** (const DiagMatrix &a, const Matrix &b)  
— : Matrix **operator \*** (const DiagMatrix &a, const Matrix &b)

— : ComplexMatrix **operator +** (const DiagMatrix &a, const ComplexMatrix &b)  
— : ComplexMatrix **operator -** (const DiagMatrix &a, const ComplexMatrix &b)  
— : ComplexMatrix **operator \*** (const DiagMatrix &a, const ComplexMatrix &b)

— : ColumnVector **diag** (void) const  
— : ColumnVector **diag** (int k) const

— : ostream& **operator <<** (ostream &os, const DiagMatrix &a)

— : **ComplexMatrix** (void)  
— : **ComplexMatrix** (int r, int c)  
— : **ComplexMatrix** (int r, int c, const Complex &val)  
— : **ComplexMatrix** (const Matrix &a)  
— : **ComplexMatrix** (const Array2<Complex> &a)  
— : **ComplexMatrix** (const ComplexMatrix &a)  
— : **ComplexMatrix** (const DiagMatrix &a)  
— : **ComplexMatrix** (const DiagArray<Complex> &a)  
— : **ComplexMatrix** (const ComplexDiagMatrix &a)

— : ComplexMatrix& **operator =** (const ComplexMatrix &a)

— : int **operator ==** (const ComplexMatrix &a) const  
— : int **operator !=** (const ComplexMatrix &a) const

— : ComplexMatrix& **insert** (const Matrix &a, int r, int c)  
— : ComplexMatrix& **insert** (const RowVector &a, int r, int c)  
— : ComplexMatrix& **insert** (const ColumnVector &a, int r, int c)  
— : ComplexMatrix& **insert** (const DiagMatrix &a, int r, int c)

— : ComplexMatrix& **insert** (const ComplexMatrix &a, int r, int c)  
— : ComplexMatrix& **insert** (const ComplexRowVector &a, int r, int c)  
— : ComplexMatrix& **insert** (const ComplexColumnVector &a, int r, int c)  
— : ComplexMatrix& **insert** (const ComplexDiagMatrix &a, int r, int c)

— : ComplexMatrix& **fill** (double val)  
— : ComplexMatrix& **fill** (const Complex &val)  
— : ComplexMatrix& **fill** (double val, int r1, int c1, int r2, int c2)  
— : ComplexMatrix& **fill** (const Complex &val, int r1, int c1, int r2, int c2)

— : ComplexMatrix **append** (const Matrix &a) const  
— : ComplexMatrix **append** (const RowVector &a) const  
— : ComplexMatrix **append** (const ColumnVector &a) const  
— : ComplexMatrix **append** (const DiagMatrix &a) const

— : ComplexMatrix **append** (const ComplexMatrix &a) const  
— : ComplexMatrix **append** (const ComplexRowVector &a) const  
— : ComplexMatrix **append** (const ComplexColumnVector &a) const  
— : ComplexMatrix **append** (const ComplexDiagMatrix &a) const

— : ComplexMatrix **stack** (const Matrix &a) const  
— : ComplexMatrix **stack** (const RowVector &a) const  
— : ComplexMatrix **stack** (const ColumnVector &a) const  
— : ComplexMatrix **stack** (const DiagMatrix &a) const

— : ComplexMatrix **stack** (const ComplexMatrix &a) const  
— : ComplexMatrix **stack** (const ComplexRowVector &a) const  
— : ComplexMatrix **stack** (const ComplexColumnVector &a) const  
— : ComplexMatrix **stack** (const ComplexDiagMatrix &a) const

— : ComplexMatrix **transpose** (void) const

— : Matrix **real** (const ComplexMatrix &a)  
— : Matrix **imag** (const ComplexMatrix &a)  
— : ComplexMatrix **conj** (const ComplexMatrix &a)

— : ComplexMatrix **extract** (int r1, int c1, int r2, int c2) const

— : ComplexRowVector **row** (int i) const  
— : ComplexRowVector **row** (char \*s) const

— : ComplexColumnVector **column** (int i) const  
— : ComplexColumnVector **column** (char \*s) const

— : ComplexMatrix **inverse** (void) const  
— : ComplexMatrix **inverse** (int &info) const  
— : ComplexMatrix **inverse** (int &info, double &rcond) const

— : ComplexMatrix **fourier** (void) const  
— : ComplexMatrix **ifourier** (void) const

— : ComplexDET **determinant** (void) const  
— : ComplexDET **determinant** (int &info) const  
— : ComplexDET **determinant** (int &info, double &rcond) const

— : ComplexMatrix **solve** (const Matrix &b) const  
— : ComplexMatrix **solve** (const Matrix &b, int &info) const  
— : ComplexMatrix **solve** (const Matrix &b, int &info, double &rcond) const

— : ComplexMatrix **solve** (const ComplexMatrix &b) const  
— : ComplexMatrix **solve** (const ComplexMatrix &b, int &info) const  
— : ComplexMatrix **solve** (const ComplexMatrix &b, int &info, double &rcond) const

— : ComplexColumnVector **solve** (const ComplexColumnVector &b) const  
— : ComplexColumnVector **solve** (const ComplexColumnVector &b, int &info) const  
— : ComplexColumnVector **solve** (const ComplexColumnVector &b, int &info, double &rcond) const

— : ComplexMatrix **lssolve** (const ComplexMatrix &b) const  
— : ComplexMatrix **lssolve** (const ComplexMatrix &b, int &info) const  
— : ComplexMatrix **lssolve** (const ComplexMatrix &b, int &info, int &rank) const

— : ComplexColumnVector **lssolve** (const ComplexColumnVector &b) const  
— : ComplexColumnVector **lssolve** (const ComplexColumnVector &b, int &info) const  
— : ComplexColumnVector **lssolve** (const ComplexColumnVector &b, int &info, int &rank) const

— : ComplexMatrix& **operator +=** (const DiagMatrix &a)  
— : ComplexMatrix& **operator -=** (const DiagMatrix &a)

— : ComplexMatrix& **operator +=** (const ComplexDiagMatrix &a)  
— : ComplexMatrix& **operator -=** (const ComplexDiagMatrix &a)

— : ComplexMatrix& **operator +=** (const Matrix &a)  
— : ComplexMatrix& **operator -=** (const Matrix &a)

— : ComplexMatrix& **operator +=** (const ComplexMatrix &a)  
— : ComplexMatrix& **operator -=** (const ComplexMatrix &a)

— : Matrix **operator !** (void) const

— : ComplexMatrix **operator +** (const ComplexMatrix &a, double s)  
— : ComplexMatrix **operator -** (const ComplexMatrix &a, double s)  
— : ComplexMatrix **operator \*** (const ComplexMatrix &a, double s)  
— : ComplexMatrix **operator /** (const ComplexMatrix &a, double s)

— : ComplexMatrix **operator +** (double s, const ComplexMatrix &a)  
— : ComplexMatrix **operator -** (double s, const ComplexMatrix &a)  
— : ComplexMatrix **operator \*** (double s, const ComplexMatrix &a)  
— : ComplexMatrix **operator /** (double s, const ComplexMatrix &a)

— : ComplexColumnVector **operator \*** (const ComplexMatrix &a, const ColumnVector &b)

— : ComplexColumnVector **operator \*** (const ComplexMatrix &a, const ComplexColumnVector &b)

— : ComplexMatrix **operator +** (const ComplexMatrix &a, const DiagMatrix &b)  
— : ComplexMatrix **operator -** (const ComplexMatrix &a, const DiagMatrix &b)  
— : ComplexMatrix **operator \*** (const ComplexMatrix &a, const DiagMatrix &b)

— : ComplexMatrix **operator +** (const ComplexMatrix &a, const ComplexDiagMatrix &b)  
— : ComplexMatrix **operator -** (const ComplexMatrix &a, const ComplexDiagMatrix &b)  
— : ComplexMatrix **operator \*** (const ComplexMatrix &a, const ComplexDiagMatrix &b)

— : ComplexMatrix **operator +** (const ComplexMatrix &a, const Matrix &b)  
— : ComplexMatrix **operator -** (const ComplexMatrix &a, const Matrix &b)

— : ComplexMatrix **operator \*** (const ComplexMatrix &a, const Matrix &b)  
— : ComplexMatrix **operator \*** (const ComplexMatrix &a, const ComplexMatrix &b)

— : ComplexMatrix **product** (const ComplexMatrix &a, const Matrix &b)  
— : ComplexMatrix **quotient** (const ComplexMatrix &a, const Matrix &b)

— : ComplexMatrix **map** (c\_c\_Mapper f, const ComplexMatrix &a)  
— : Matrix **map** (d\_c\_Mapper f, const ComplexMatrix &a)  
— : void **map** (c\_c\_Mapper f)

— : Matrix **all** (void) const  
— : Matrix **any** (void) const

— : ComplexMatrix **cumprod** (void) const  
— : ComplexMatrix **cumsum** (void) const  
— : ComplexMatrix **prod** (void) const  
— : ComplexMatrix **sum** (void) const  
— : ComplexMatrix **sumsq** (void) const

— : ComplexColumnVector **diag** (void) const  
— : ComplexColumnVector **diag** (int k) const

— : ComplexColumnVector **row\_min** (void) const  
— : ComplexColumnVector **row\_min\_loc** (void) const

— : ComplexColumnVector **row\_max** (void) const  
— : ComplexColumnVector **row\_max\_loc** (void) const

— : ComplexRowVector **column\_min** (void) const  
— : ComplexRowVector **column\_min\_loc** (void) const

— : ComplexRowVector **column\_max** (void) const  
— : ComplexRowVector **column\_max\_loc** (void) const

— : ostream& **operator <<** (ostream &os, const ComplexMatrix &a)  
— : istream& **operator >>** (istream &is, ComplexMatrix &a)

— : **ComplexColumnVector** (void)  
— : **ComplexColumnVector** (int n)  
— : **ComplexColumnVector** (int n, const Complex &val)  
— : **ComplexColumnVector** (const ColumnVector &a)  
— : **ComplexColumnVector** (const Array<Complex> &a)  
— : **ComplexColumnVector** (const ComplexColumnVector &a)

— : ComplexColumnVector& **operator =** (const ComplexColumnVector &a)

— : int **operator ==** (const ComplexColumnVector &a) const  
— : int **operator !=** (const ComplexColumnVector &a) const

— : ComplexColumnVector& **insert** (const ColumnVector &a, int r)  
— : ComplexColumnVector& **insert** (const ComplexColumnVector &a, int r)

— : ComplexColumnVector& **fill** (double val)  
— : ComplexColumnVector& **fill** (const Complex &val)  
— : ComplexColumnVector& **fill** (double val, int r1, int r2)  
— : ComplexColumnVector& **fill** (const Complex &val, int r1, int r2)

— : ComplexColumnVector **stack** (const ColumnVector &a) const  
— : ComplexColumnVector **stack** (const ComplexColumnVector &a) const

— : ComplexRowVector **transpose** (void) const

— : ColumnVector **real** (const ComplexColumnVector &a)  
— : ColumnVector **imag** (const ComplexColumnVector &a)  
— : ComplexColumnVector **conj** (const ComplexColumnVector &a)

— : ComplexColumnVector **extract** (int r1, int r2) const

— : ComplexColumnVector& **operator +=** (const ColumnVector &a)  
— : ComplexColumnVector& **operator -=** (const ColumnVector &a)

— : ComplexColumnVector& **operator +=** (const ComplexColumnVector &a)  
— : ComplexColumnVector& **operator -=** (const ComplexColumnVector &a)

— : ComplexColumnVector **operator +** (const ComplexColumnVector &a, double s)  
— : ComplexColumnVector **operator -** (const ComplexColumnVector &a, double s)  
— : ComplexColumnVector **operator \*** (const ComplexColumnVector &a, double s)  
— : ComplexColumnVector **operator /** (const ComplexColumnVector &a, double s)

— : ComplexColumnVector **operator +** (double s, const ComplexColumnVector &a)  
— : ComplexColumnVector **operator -** (double s, const ComplexColumnVector &a)  
— : ComplexColumnVector **operator \*** (double s, const ComplexColumnVector &a)  
— : ComplexColumnVector **operator /** (double s, const ComplexColumnVector &a)

— : ComplexMatrix **operator \*** (const ComplexColumnVector &a, const ComplexRowVector &b)

— : ComplexColumnVector **operator +** (const ComplexColumnVector &a, const ColumnVector &b)  
— : ComplexColumnVector **operator -** (const ComplexColumnVector &a, const ColumnVector &b)

— : ComplexColumnVector **product** (const ComplexColumnVector &a, const ColumnVector &b)  
— : ComplexColumnVector **quotient** (const ComplexColumnVector &a, const ColumnVector &b)

— : ComplexColumnVector **map** (c\_c\_Mapper f, const ComplexColumnVector &a)  
— : ColumnVector **map** (d\_c\_Mapper f, const ComplexColumnVector &a)  
— : void **map** (c\_c\_Mapper f)

— : Complex **min** (void) const  
— : Complex **max** (void) const

— : ostream& **operator <<** (ostream &os, const ComplexColumnVector &a)

— : **ComplexRowVector** (void)  
— : **ComplexRowVector** (int n)  
— : **ComplexRowVector** (int n, const Complex &val)  
— : **ComplexRowVector** (const RowVector &a)  
— : **ComplexRowVector** (const Array<Complex> &a)  
— : **ComplexRowVector** (const ComplexRowVector &a)

— : ComplexRowVector& **operator =** (const ComplexRowVector &a)

— : int **operator ==** (const ComplexRowVector &a) const  
— : int **operator !=** (const ComplexRowVector &a) const

— : ComplexRowVector& **insert** (const RowVector &a, int c)  
— : ComplexRowVector& **insert** (const ComplexRowVector &a, int c)

— : ComplexRowVector& **fill** (double val)  
— : ComplexRowVector& **fill** (const Complex &val)  
— : ComplexRowVector& **fill** (double val, int c1, int c2)  
— : ComplexRowVector& **fill** (const Complex &val, int c1, int c2)

— : ComplexRowVector **append** (const RowVector &a) const  
— : ComplexRowVector **append** (const ComplexRowVector &a) const

— : ComplexColumnVector **transpose** (void) const

— : RowVector **real** (const ComplexRowVector &a)  
— : RowVector **imag** (const ComplexRowVector &a)  
— : ComplexRowVector **conj** (const ComplexRowVector &a)

— : ComplexRowVector **extract** (int c1, int c2) const

— : ComplexRowVector& **operator +=** (const RowVector &a)  
— : ComplexRowVector& **operator -=** (const RowVector &a)

— : ComplexRowVector& **operator +=** (const ComplexRowVector &a)  
— : ComplexRowVector& **operator -=** (const ComplexRowVector &a)

— : ComplexRowVector **operator +** (const ComplexRowVector &a, double s)  
— : ComplexRowVector **operator -** (const ComplexRowVector &a, double s)  
— : ComplexRowVector **operator \*** (const ComplexRowVector &a, double s)  
— : ComplexRowVector **operator /** (const ComplexRowVector &a, double s)

— : ComplexRowVector **operator +** (double s, const ComplexRowVector &a)  
— : ComplexRowVector **operator -** (double s, const ComplexRowVector &a)  
— : ComplexRowVector **operator \*** (double s, const ComplexRowVector &a)  
— : ComplexRowVector **operator /** (double s, const ComplexRowVector &a)

— : Complex **operator \*** (const ComplexRowVector &a, const ColumnVector &b)

— : Complex **operator \*** (const ComplexRowVector &a, const ComplexColumnVector &b)

— : ComplexRowVector **operator \*** (const ComplexRowVector &a, const ComplexMatrix &b)

— : ComplexRowVector **operator +** (const ComplexRowVector &a, const RowVector &b)  
— : ComplexRowVector **operator -** (const ComplexRowVector &a, const RowVector &b)

— : ComplexRowVector **product** (const ComplexRowVector &a, const RowVector &b)  
— : ComplexRowVector **quotient** (const ComplexRowVector &a, const RowVector &b)

— : ComplexRowVector **map** (c\_c\_Mapper f, const ComplexRowVector &a)  
— : RowVector **map** (d\_c\_Mapper f, const ComplexRowVector &a)  
— : void **map** (c\_c\_Mapper f)

— : Complex **min** (void) const  
— : Complex **max** (void) const

— : ostream& **operator <<** (ostream &os, const ComplexRowVector &a)

— : **ComplexDiagMatrix** (void)  
— : **ComplexDiagMatrix** (int n)  
— : **ComplexDiagMatrix** (int n, const Complex &val)  
— : **ComplexDiagMatrix** (int r, int c)  
— : **ComplexDiagMatrix** (int r, int c, const Complex &val)  
— : **ComplexDiagMatrix** (const RowVector &a)  
— : **ComplexDiagMatrix** (const ComplexRowVector &a)  
— : **ComplexDiagMatrix** (const ColumnVector &a)  
— : **ComplexDiagMatrix** (const ComplexColumnVector &a)  
— : **ComplexDiagMatrix** (const DiagMatrix &a)  
— : **ComplexDiagMatrix** (const DiagArray<Complex> &a)  
— : **ComplexDiagMatrix** (const ComplexDiagMatrix &a)

— : ComplexDiagMatrix& **operator =** (const ComplexDiagMatrix &a)

— : int **operator ==** (const ComplexDiagMatrix &a) const  
— : int **operator !=** (const ComplexDiagMatrix &a) const

— : ComplexDiagMatrix& **fill** (double val)  
— : ComplexDiagMatrix& **fill** (const Complex &val)  
— : ComplexDiagMatrix& **fill** (double val, int beg, int end)  
— : ComplexDiagMatrix& **fill** (const Complex &val, int beg, int end)  
— : ComplexDiagMatrix& **fill** (const ColumnVector &a)  
— : ComplexDiagMatrix& **fill** (const ComplexColumnVector &a)  
— : ComplexDiagMatrix& **fill** (const RowVector &a)  
— : ComplexDiagMatrix& **fill** (const ComplexRowVector &a)  
— : ComplexDiagMatrix& **fill** (const ColumnVector &a, int beg)  
— : ComplexDiagMatrix& **fill** (const ComplexColumnVector &a, int beg)  
— : ComplexDiagMatrix& **fill** (const RowVector &a, int beg)  
— : ComplexDiagMatrix& **fill** (const ComplexRowVector &a, int beg)

— : ComplexDiagMatrix **transpose** (void) const

— : DiagMatrix **real** (const ComplexDiagMatrix &a)  
— : DiagMatrix **imag** (const ComplexDiagMatrix &a)  
— : ComplexDiagMatrix **conj** (const ComplexDiagMatrix &a)

— : ComplexMatrix **extract** (int r1, int c1, int r2, int c2) const

— : ComplexRowVector **row** (int i) const  
— : ComplexRowVector **row** (char \*s) const

— : ComplexColumnVector **column** (int i) const  
— : ComplexColumnVector **column** (char \*s) const

— : ComplexDiagMatrix **inverse** (int &info) const  
— : ComplexDiagMatrix **inverse** (void) const

— : ComplexDiagMatrix& **operator +=** (const DiagMatrix &a)  
— : ComplexDiagMatrix& **operator -=** (const DiagMatrix &a)

— : ComplexDiagMatrix& **operator +=** (const ComplexDiagMatrix &a)  
— : ComplexDiagMatrix& **operator -=** (const ComplexDiagMatrix &a)

— : ComplexMatrix **operator +** (const ComplexDiagMatrix &a, double s)  
— : ComplexMatrix **operator -** (const ComplexDiagMatrix &a, double s)

— : ComplexMatrix **operator +** (const ComplexDiagMatrix &a, const Complex &s)  
— : ComplexMatrix **operator -** (const ComplexDiagMatrix &a, const Complex &s)

— : ComplexDiagMatrix **operator \*** (const ComplexDiagMatrix &a, double s)  
— : ComplexDiagMatrix **operator /** (const ComplexDiagMatrix &a, double s)

— : ComplexMatrix **operator +** (double s, const ComplexDiagMatrix &a)  
— : ComplexMatrix **operator -** (double s, const ComplexDiagMatrix &a)

— : ComplexMatrix **operator +** (const Complex &s, const ComplexDiagMatrix &a)  
— : ComplexMatrix **operator -** (const Complex &s, const ComplexDiagMatrix &a)

— : ComplexDiagMatrix **operator \*** (double s, const ComplexDiagMatrix &a)

— : ComplexColumnVector **operator \*** (const ComplexDiagMatrix &a, const ColumnVector &b)

— : ComplexColumnVector **operator \*** (const ComplexDiagMatrix &a, const ComplexColumnVector &b)

— : ComplexDiagMatrix **operator +** (const ComplexDiagMatrix &a, const DiagMatrix &b)  
— : ComplexDiagMatrix **operator -** (const ComplexDiagMatrix &a, const DiagMatrix &b)

— : ComplexDiagMatrix **product** (const ComplexDiagMatrix &a, const DiagMatrix &b)

— : ComplexMatrix **operator +** (const ComplexDiagMatrix &a, const Matrix &b)  
— : ComplexMatrix **operator -** (const ComplexDiagMatrix &a, const Matrix &b)  
— : ComplexMatrix **operator \*** (const ComplexDiagMatrix &a, const Matrix &b)

— : ComplexMatrix **operator +** (const ComplexDiagMatrix &a, const ComplexMatrix &b)  
— : ComplexMatrix **operator -** (const ComplexDiagMatrix &a, const ComplexMatrix &b)  
— : ComplexMatrix **operator \*** (const ComplexDiagMatrix &a, const ComplexMatrix &b)

— : ComplexColumnVector **diag** (void) const  
— : ComplexColumnVector **diag** (int k) const

— : ostream& **operator <<** (ostream &os, const ComplexDiagMatrix &a)
